# Anticancer activity of *Ilex khasiana*, a rare and endemic species of holly in Northeast India, against murine lymphoma

**DOI:** 10.1016/j.heliyon.2025.e41839

**Published:** 2025-01-10

**Authors:** Charles Lalnunfela, Pawi Bawitlung Lalthanpuii, Hmar Tlawmte Lalremsanga, Zothansiama, Chhaihlo Lalmuansangi, Mary Zosangzuali, Nachimuthu Senthil Kumar, Tochhawng Lalhriatpuii, Kholhring Lalchhandama

**Affiliations:** a Department of Zoology, Mizoram University, Tanhril, 796004, Mizoram, India; b DBT-BUILDER National Laboratory, Department of Life Sciences, Pachhunga University College, Aizawl, 796001, Mizoram, India; c Department of Biotechnology, Mizoram University, Tanhril, 796004, Mizoram, India; d Department of Pharmacy, Regional Institute of Paramedical and Nursing Sciences, Zemabawk, 796017, Mizoram, India

**Keywords:** Anticancer activity, Lymphoma, Mizo traditional medicine, Molecular docking

## Abstract

*Ilex khasiana* Purkay. is a lesser-known species of holly (family Aquifoliaceae) that is endemic to Northeast India. Designated as critically endangered, the plant is used in the treatments of bacterial infections, cancer, intestinal helminthiasis, tuberculosis, and viral infections. A methanol extract of the leaves was prepared from which 16 different compounds were identified using gas chromatography-mass spectroscopy. An alkylated phenol, 2,6-di-tert-butylphenol, was the predominant compound. Acute toxicity test indicated that the plant extract was non-toxic even at the highest dosage tested, i.e., 2000 mg/kg body weight. The plant extract caused considerable prolongation of survival in mice transplanted with Dalton's lymphoma ascites, extending life by 33 %, with median survival time of 35.5 and average survival time of 22.83 days, and with a treatment to control ratio of 131.37 %. Reduction of body mass, lipid peroxidation, alanine transaminase, aspartate aminotransferase, and creatinine were seen in DLA-transplanted mice after treatment with the plant extract. On the other hand, glutathione level, glutathione S-transferase and superoxide dismutase activity increased. Alkaline comet assay showed that the plant extract effectively induced DNA damage, producing a tail length of 11.89 μm and Olive moment of 2.36 at 250 mg/kg bwt, the most effective dosage. Molecular docking revealed high ligand binding ability of 2,6-di-tert-butylphenol to chemokine receptor CXCR4, DNA topoisomerase 2-alpha, DNA topoisomerase 2-beta, histone deacetylases (HDAC1, HDAC2, HDAC3), Janus kinase 1 and programmed cell death protein 1. The safety and anticancer activity in the present study substantiate the therapeutic importance of *I. khasiana* as acclaimed in the Mizo traditional medicine. Additionally, the study advocates further pharmacological investigations as well as the conservation and propagation of the endangered plant for future research.

## Highlights

• *Ilex khasiana* is a critically endangered holly in Northeast India and is a traditional therapy for helminthiasis and cancer, among other.
• The leaf extract showed anticancer activity against Dalton's lymphoma ascites in mice.
• Its predominant compound, 2,6-di-tert-butylphenol has high binding energy on several receptors implicated to lymphoma.

## 1 Introduction

Cancer is a contingent of diseases arising from interrupted cell cycle machinery due to varied carcinogens from exogenous substances, radiations, viruses, parasites, autoimmune disorders and lifestyle habits. As a multifactorial disease, the underpinning cellular and molecular events are highly variable among the types of cancers [1]. Described as the universal six hallmarks, the quintessential blueprint of cancer traits consists of sustained proliferation, dodging growth inhibitors, evading normal cell death, capability of unlimited replication, invasive cellular effusion and actuating unchecked interphase in the otherwise normal cells [2]. The interwoven molecular complexities are the factors behind a sustained hindrance in cancer therapy and enduring high death toll in spite of cancer biology at the forefront of global research [3]. Many drugs have been introduced in cancer management, but each of which is manifested to be associated with critical drawbacks, specifically involving the developments of life-threatening adverse effects or lasting morbidity [4]. The problem is no less compounded by another characteristic of cancer cells – they develop drug resistance with relative ease, rendering the available therapeutics systematically ineffective. The need for new and improved medications is demanding as ever [5].

Medicinal plants have provided some of the best pharmaceutical drugs in cancer therapy and novel lead molecules for drug developments [6]. The vinca alkaloids such as vinblastine and vincristine, a tetracyclic diterpenoid paclitaxel, and an indole alkaloid camptothecin, including their various derivatives, remain the mainstay of cancer medications [7]. A number of phytocompounds are under clinical investigations, including polyphenols such as curcumin, resveratrol and epigallocatechin gallate [8], flavonoids like quercetin and kaempferol, sesquiterpene lactone like artemisinin, pentacyclic triterpene like betulinic acid, phenol like gingerol, and glycoside like rutin [6]. Study of medicinal plants for anticancer activity is thus one of the principal keystones in medicine and pharmacology, with several species under promising investigations [9].

Commonly known as holly, *Ilex* Tourn. ex L. constitutes the only extant genus in the family Aquifoliaceae and an interesting group of plants in Asian and South American traditional medicines [10]. Species like *I. kudingcha* C.J. Tseng and *I. latifolia* Thunb. are widely used in traditional Chinese medicine for the treatments of blood diseases, body pain, diarrhoea, fever, and liver damages [11]. *I. pubescens* Hook. & Arn. is used as a remedy for asthma, coronary heart diseases, hepatitis, hyperlipidaemia, and hypertension [12]. Pharmacological tests have indicated the anticancer [13], analgesic, anti-inflammatory [14], anticoagulant [15], and neuro-protective activities of the bioactive components [16]. In several South American cultures, *I. paraguariensis* A. St.-Hil. is taken as a herbal tea, and is therapeutically used as cancer remedy, anti-obesity diet and anti-inflammatory agent [17]. The plant's anticancer property has been experimentally ascertained against various cell lines [18,19].

*I. khasiana* Purkay. is a large tree that has been categorised as critically endangered according to the World Conservation Monitoring Centre and then included in the IUCN Red List of *Threatened Species* [20]. Endemic to Northeast India, it was first identified from the Khasi Hills in Meghalaya, and later recorded in Aizawl, Mizoram [21]. Only few individual trees are growing in these regions. Its various ethnomedicinal uses for different diseases have been documented [21,22]. The Khasi people used the plant extract as an antibacterial agent in tuberculosis and antiviral in cold and fever. The leaves are consumed for the treatments of bacterial and parasitic infections, cancer and blood diseases in the Mizo traditional medicine. Following the acclaimed therapeutic applications, the antioxidant [23], antiparasitic [24], anti-inflammatory and analgesic activities have been evaluated and reported [25]. However, its purported anticancer property remains unexplored, upon which analysis of the chemical components and pre-clinical experiments were performed to have an insight on the anticancer effects of the plant.

## 2 Materials and methods

### 2.1 Plant specimen and extraction

The leaves of *I*. *khasiana* were collected from Luangmual, Aizawl, Mizoram, India, located at 23°44.556′N and 92°41.956′E. The voucher herbarium was validated at the Botanical Survey of India, Eastern Regional Office, Shillong, Meghalaya. It was deposited with an accession number BSI/EC/Tech./2008/577 at the Department of Pharmacy Herbarium Section of the Regional Institute of Paramedical and Nursing Sciences, Mizoram, India. Additional authentication was done with specimens at the Royal Botanic Gardens, Kew (taxon code 83411-1 on https://powo.science.kew.org) and that of The Plant List (taxon code 2860918 on http://www.theplantlist.org). After washing the leaves with distilled water, they were left to dry in the shade at a room temperature of 24 ± 1 °C. The dried samples were pulverised in a grinder to produce fine powder. Extraction was performed in a Soxhlet apparatus of 5-L capacity using methanol as a solvent and was run continuously for three days. A semi-solid crude extract was collected and concentrated by removing and recovering the solvent in a pressurised rotary vacuum evaporator (Buchi Rotavapor® R-215, Flawil, Switzerland). The final extract was stored at 4 °C.

### 2.2 Chemicals

All chemicals were either standard analytical or chromatographic grades. Ammonium oxalate, hydrochloric acid, disodium biphosphate, methanol (HPLC grade), n-butanol, sodium chloride, sodium hydroxide, sulphuric acid and Tris buffer were products of Merck India, Mumbai. Hydrogen peroxide, potassium chloride, and trichloroacetic acid were procured from SD Fine-Chem Limited, Mumbai, India. Coomassie brilliant blue, methanol and nicotinamide adenine dinucleotide (NADH) were supplied by HiMedia Private Limited, Mumbai, India. Doxorubicin was obtained from Getwell Pharmaceuticals, Gurgaon, India. All other chemicals were supplied from Sigma-Aldrich Chemicals Private Limited, Kolkata, India.

### 2.3 Chemical profiling

Chemical compounds in *I*. *khasiana* extract were analysed in a single quadrupole gas chromatography-mass spectrometry TRACE™ 1300 ISQ™ LT (Thermo Scientific, Waltham, USA). A column, TR-5MS (dimension 30 m × 0.25 mm × 0.25 μm with film thickness of 0.25 μm) was used as a stationary phase. The carrier gas was helium that was injected at a rate of 1 mL/min in a splitting ratio of 1:50. Temperature was set to 250 °C for the transfer line and to 220 °C for the ion-source line. The mass spectrometer was run for 32 min. Chemical data were generated from Thermo Scientific™ Xcalibur™ software and compounds were identified based on their chemical formulae, retention times and molecular weights from the libraries of Wiley Registry™ and National Institute of Standards and Technology.

### 2.4 Maintenance of mice

Swiss albino mice were used for experimentations as approved by the Institutional Animal Ethics Committee of the Regional Institute of Paramedical and Nursing Sciences (approval number IAEC/RIPANS/27). The mice were reared in polyvinyl cages under regulated gaseous atmosphere at a temperature of 25 ± 1 °C, with 50 % humidity in 12 h light and dark cycle at the Institutional Animal House of RIPANS. They were provided free access to water and food pellets *ad libitum.* Individuals of both sexes in balanced ratio, 10–12 weeks old, each weighing 23 ± 2 g were chosen for the *in vivo* assays.

### 2.5 Acute toxicity

The oral acute toxicity of *I. khasiana* extract was assessed according to the guidelines of the Organization for Economic Co-operation and Development (OECD, 2002) [26]. Five sets of mice, each of three male and three female, were fasted for 4 h, providing water *ad libitum* for each of the control and experimental groups. Control group continued to receive only water. The test groups were then fed *I. khasiana* extract using feeding tubes in single dosage in three doses, i.e., 5, 50, 300 and 2000 mg/kg body weight (bwt). The animals were observed for 14 days for any behavioural change. Toxicity was defined as signs of discomfort or mortality in at least half of the treated animals before the end of the observation.

### 2.6 Tumour induction and evaluation of longevity and body mass

Mice were divided into five groups, each with six individuals of both sexes in equal numbers. Malignant and aggressive tumour cells, Dalton's lymphoma ascites (DLA) were administered by intraperitoneal injection at a concentration of 1 × 10^6^ cells suspended in 250 μL phosphate-buffered saline (PBS) to each mice. Seven groups of treatments were given after 48 h of complete transplantation: Group I were given only PBS at 10 mL/kg bwt and maintained as a negative control; Group II received doxorubicin at 0.5 mg/kg bwt as a positive control; Group III to V were given *I. khasiana* extracts at 100, 250 and 500 mg/kg bwt respectively. Daily treatments were continued for seven days. After the first day of treatment, mice from each group were weighed every third day. The anti-tumour responses were determined from the median survival time (MST) and average survival time (AST) of the mice. From values of the survival times, percentage increase in median life span (IMLS) and percentage increase in average life span (IALS) were also quantified [27]. The overall anticancer activity was evaluated from the treatment to control (T/C) ratio, the value of which was estimated from the equation:

T/C = [MST of treated group ÷ MST of control]

### 2.7 Preparation of tissue samples

Another seven groups of mice were maintained and given treatments similarly as before. Upon completion of the treatments, they were sacrificed by inducing euthanasia with an overdose of ketamine as recommended [28]. On autopsy, the liver and intraperitoneal lysate were collected from each experimental mouse. The liver homogenates in 5 % (w/v) were prepared using a glass homogeniser in an ice-cold buffer consisting of 5 mM ethylenediaminetetraacetic acid (EDTA) and 0.15 M sodium chloride (NaCl) at pH 7.4. They were centrifuged at 13,000 rpm and 4 °C for 30 min. The resultant supernatant was collected and immediately stored at −20 °C. The intraperitoneal lysate was obtained by repeatedly washing the intraperitoneal fluid with ammonium chloride (NH_4_Cl) and 1X PBS. To get 5 % (w/v) homogenate, the cells were sonicated in EDTA + NaCl buffer as in the liver homogenates. After centrifugation at 10,000 rpm at 4 °C for 30 min, a supernatant was formed that was immediately stored at −20 °C.

### 2.8 Detoxification assay

Tissue homogenate and intraperitoneal lysate were first estimated for the total protein contents using the classic Folin-Ciocalteau reaction method [29]. Glutathione (GSH) was determined from Ellman's reaction based on a thiol exchange between a reactant and 5,5-dithio-bis-(2-nitrobenzoic acid) [30]. The optical density (OD) of the yellow solution was detected at the wavelength of 412 nm in a UV–visible double-beam spectrophotometer. The amount of GSH was calculated from the standard graph. Determination of glutathione S-transferase (GST) activity was done from 1-chloro-2,4-dinitrobenzene (CDNB) reaction [31]. A reactant mixture of 0.1 mL of 20 mM CDNB, 0.5 mL of 0.1 M phosphate buffer (pH 6.5) and 8.8 mL distilled were first incubated at 30 °C for 10 min. Then, 0.5 mL of 20 mM GSH was added to 0.1 mL each of the liver and intraperitoneal lysate samples and were mixed with the reactant mixture till coloured solutions were formed. The intensity of the coloured solution was measured at 340 nm at 1 min intervals for 6 min. The total GST activity was determined form the equation:

GST activity = [OD of sample – OD of blank] × [1000 ÷ 9.6] × Volume of sample

Where 9.6 represents the molar extinction coefficient of GST.

### 2.9 Superoxide dismutase assay

Superoxide dismutase (SOD) activity was estimated using nitroblue tetrazolium (NBT) reduction test [32]. 100 μL of the liver and intraperitoneal lysate samples were incubated at 30 °C after adding a mixture of 300 μL of 3.0 mM NBT, 200 μL of 780 μM NADH and 100 μL of 186 μM phenazene methosulfate. The reaction was terminated at 90 min by adding 1 mL of acetic acid followed by 4 mL n-butanol. The OD was taken at 560 nm. The enzyme activity was estimated from the equation:

SOD activity = [(OD of blank – OD of sample) ÷ OD of blank] × 100.

The enzyme activity was expressed as 50 % inhibition of reduced NBT per mg protein.

### 2.10 Lipid peroxidation assay

Lipid peroxidation was determined following the fluorescence reaction method of Buege and Aust [33]. Basically, malondialdehyde (MDA) is one of the end products of the oxidations of polyunsaturated fatty acids. MDA is highly reactive and readily gives red fluorescence upon reacting with thiobarbituric acid. The intensity of colour was detected at 535 nm. The amounts of MDA in the liver and intraperitoneal lysate samples were calculated from the extinction coefficient of 1.56 × 10^6^ M^−1^ cm^−1^.

### 2.11 Haematological tests

Serum levels of liver enzymes like alanine transaminase and aspartate aminotransferase, and a kidney waste, creatinine are diagnostic indicators of metabolic disorders including cancer. For haematological tests, mice were grouped and given treatments as in the induction of tumour. After seven days of treatment, the animals were anaesthetised with ketamine. Using capillary tubes, blood samples were drawn from the retro-orbital sinuses and were collected at different time intervals. The blood samples were stored at 4 °C. To determine the levels of the enzymes and creatinine, the blood samples were centrifuged at 1000 rpm for 5 min and processed for colorimetric estimation using standard kits according to the manufacturer's description (Coral Clinical Systems, Uttarakhand, India).

### 2.12 Comet assay

DNA damage indicated by strand breakage was studied using alkaline single-cell gel electrophoresis or the comet assay [34]. Dalton's lymphoma ascites from both the treated and control mice were aspirated and washed with 1X PBS and NH_4_Cl. Cells at a concentration of 2 × 10^4^ were suspended in 0.5 % low-melting-point agarose (LMPA) made in 1X PBS. After trypsinisation and centrifugation at 1250 rpm for 5 min, the cells were resuspended. 75 μL of the suspension fluid was spread on 1 % normal-melting-point agarose (NMPA) frosted and precoated slides. The slides were covered with cover slips. The cover slips were opened to add 90 μL 0.5 % LMPA, closed again and then incubated at 4 °C for 4 h. The slides were immersed in a lysis buffer composed of 10 % dimethyl sulfoxide, 100 mM Na_2_EDTA, 2.5 M NaCl, 1 % Triton X-100 and 10 mM Trizma base (pH 10) for 2 h. The slides were transferred to an electrophoresis tank containing an alkaline buffer made up of 300 mM NaOH, 1 mM Na_2_EDTA (pH 13) for 20 min to facilitate denaturation and unwinding of the DNA strands. Electrophoresis was run at 24 V and 300 mA for 30 min. The slides were washed for 5 min in neutralising buffer consisting of 0.4 M Tris-HCl (pH 7.5), and then with distilled water. After staining for 5 min in ethidium bromide (2 μg/mL), the slides were observed under a fluorescent microscope (Invitrogen™ EVOS™ FL auto 2). Cell images were captured and analysed in Image J software (National Institutes of Health, USA) for measuring the tail lengths and Olive moments. The Olive moment was deduced from the equation:

Olive moment = (Tail mean – head mean) × % of DNA in the tail

### 2.13 Molecular docking

The three-dimensional (3D) structure of 2,6-di-tert-butylphenol was retrieved in structured data file (SDF) formats from PubChem (https://pubchem.ncbi.nlm.nih.gov), US National Center for Biotechnology Information (NCBI). ChemBio3D Ultra 12.0 (CambridgeSoft Corporation, Cambridge, USA) with a force field MMFF94 was used to optimise the structures and minimise the cumulative potential energies. 2,6-Di-tert-butylphenol was docked to proteins available at the Research Collaboratory for Structural Bioinformatics (RCSB) protein data bank (RCSB-PDB) (www.rcsb.org) including chemokine receptor CXCR4 (PDB code: 3OE6) [35], DNA topoisomerase 2-alpha (PDB code: 4FM9) [36], DNA topoisomerase 2-beta (PDB code: 3QX3 and 5ZAD) [37,38], histone deacetylase 1 (HDAC1) (PDB code: 4BKX) [39], HDAC2 (PDB code: 4LY1) [40], HDAC3 (PDB code: 4A69) [41], Janus kinase 1 (JAK1) (PDB code: 3EYG) [42], phosphoinositide 3-kinase (PI3Kɑ) (PDB code: 4JPS) [43], PI3Kβ (PDB code: 2Y3A and 4BFR) [44,45], PI3Kγ (PDB code: 4ANW) [46], and programmed cell death protein 1 (PD-1) (PDB code: 6JJP) [47]. To obtain uninterrupted configurations, molecules adhering to the targets such as co-factors, unique ligands and water were detached using Molegro Molecular Viewer software. Molecular docking was done on the AutoDock Vina v1.2.4 platform (Molecular Graphics Lab, The Scripps Research, La Jolla, USA) [48]. Kollman charges and polar hydrogens were added to the proteins using Molecular Graphics Lab biosoftware MGLTools 1.5.6 before saving the data in protein data bank, partial charge (Q) and atom type (T) (PDBQT) format.

### 2.14 Statistical analysis

Data were expressed as means ± standard error of the means. The significant difference of survival values was computed by Kaplan-Meier estimator. Variations on haematological parameters were analysed by one-way analysis of variance (ANOVA) followed by Tukey's honest significance test. Graphical data and statistics were generated using Prism GraphPad software version 10.4.0 (Dotmatics, Boston, Massachusetts, USA). Significant difference was considered at *p* value less than 0.05.

## 3 Results

### 3.1 GC-MS analysis

**Fig. 1:**
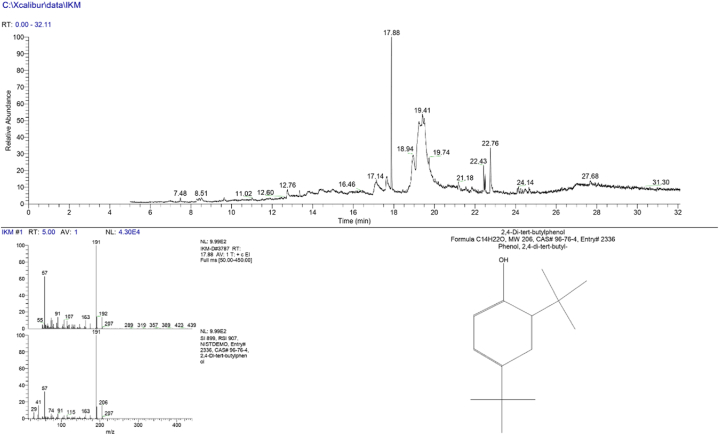
Chromatogram and mass spectra of compounds detected in *Ilex khasiana* Purkay. methanol extract (IKM) from gas chromatography-mass spectrometry.

From GC-MS chromatogram and mass spectra, 16 compounds were identified from the methanol extract of *I. khasiana* leaf. The major peaks with their relative abundance are shown in Fig. 1 and the complementary compounds resolved from chemical libraries are listed in Table 1. In general, a variety of compounds was detected including saccharides, glycosides, fatty acids, pyranones, benzenes, and a steroid. The principal chemical constituent was an alkylated phenol, 2,6-di-tert-butylphenol (C_14_H_22_O; molecular mass 206.32). Other major compounds detected were a pyranose, desulphosinigrin (C_10_H_17_NO_6_S; molecular mass 279.91) and a fatty acid, palmitic acid (C_16_H_32_O_2_; molecular mass 256.42), both of which were below the expected probability.

**Table 1:** Compounds identified from the methanol extract of the leaves of *Ilex khasiana* Purkay. (IKM) using gas chromatography-mass spectrometry.

No	Retention time (min)	Probability (%)	Compound	Chemical formula	Molecular mass
1	7.47	25.44	Chloro-(4-methoxyphenyl)-dimethylsilane	C_10_H_15_ClOSi	214.764
2	8.51	34.26	6-Oxa-bicyclo [3.1.0] hexan-3-one	C_5_H_6_O_2_	98.100
3	11.02	10.28	3,5-Di-tert-butyl-1,2-benzenediol	C_14_H_22_O_2_	222.323
4	12.60	14.81	Melezitose, monohydrate	C_18_H_32_O_16_	504.437
5	12.76	18.82	2,3-Dihydro-2,5-dihydoxy-6-methyl-4-H-pyran-4-one	C_6_H_8_O_4_	144.125
6	16.46	48.19	Desulphosinigrin	C_10_H_17_NO_6_S	279.310
7	17.14	22.03	1,4-Diacetyl-3-acetoxymethyl-2,5-methylene-l-rhamnitol	C_14_H_22_O_8_	318.131
8	17.89	72.89	2,6-Di-tert-butylphenol	C_14_H_22_O	206.320
9	18.94	12.86	Methyl-D-glucopyranoside	C_7_H_14_O_6_	194.180
10	19.41	24.28	Ethyl α-d-glucopyranoside	C_8_H_16_O_6_	208.210
11	19.74	32.12	Desulphosinigrin	C_10_H_17_NO_6_S	279.310
12	21.18	21.18	9-Octadecenoic acid, (2-phenyl-1,3-dioxolan-4-yl) methyl ester	C_28_H_44_O_4_	444.650
13	22.43	11.19	Methyl 14-Methylpentadecanoate	C_17_H_34_O_2_	270.451
14	22.76	58.85	Hexadecanoic acid/palmitic acid	C_16_H_32_O_2_	256.424
15	24.14	40.99	2′-Hexyl-1,1′-bicyclopropane-2-octanoic acid methyl ester	C_21_H_38_O_2_	322.525
16	27.68	31.68	2-Bromotetradecanoic acid	C_14_H_27_BrO_2_	306.301
17	31.30	14.90	3α-(Trimethylsiloxy)-17-(phenylmethoxyimino)-5α-androstan-11-one	C_29_H_43_NO_3_Si	481.740

### 3.2 Acute toxicity

Mice in toxicity tests thrived well after being fed with *I. khasiana* extract. After continuous observation for 14 days for any sign of discomfort and mortality, all the mice survived and displayed normal activities, indicating that they tolerated the plant extract well and that the extract had low or negligible toxicity. It is discernible that the median lethal dose (LD_50_) of the plant extract is above that of the highest dose (2000 mg/kg bwt) given, evidencing its overall safety.

### 3.3 Anticancer activity

**Fig. 2:**
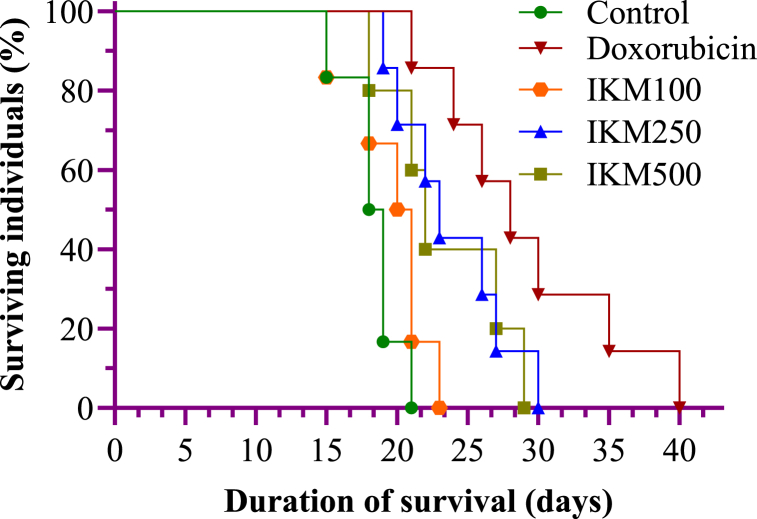
Number and duration of survival of mice transplanted with Dalton's lymphoma ascites for 40 days. Comparative survival values are constructed from Kaplan Meier's estimate. Control: tumour-bearing untreated mice; doxorubicin at 20 mg/kg bwt; IKM100, IKM250, IKM500: treatment with *Ilex khasiana* Purkay. methanol extract at the doses of 100, 250, and 500 mg/kg bwt respectively.

The survival and body mass values of mice under different experimental conditions are given in Table 2. The control group having DLA-transplanted tumour and without any treatment survived the shortest and died within four weeks of tumour induction, indicated by an MST of 25.5 days and AST of 18.3 days. Treatments with doxorubicin and *I. khasiana* extract indicated that the tumour responded well (Fig. 2). Doxorubicin was the most effective upon the treatment of which mice survived up to seven weeks (MST of 41 and AST of 28.7 days). It increased the survival time by 38 % (IMLS of 60.78 and IALS of 53.68 days) and showed high treatment-control (T/C) percentage of 160.78. Out of the three dosages of the plant extract, 250 mg/kg bwt showed the best activity, indicating an MST of 33.5 days and AST of 22.83 days, with IMLS of 31.37, IALS of 24.55 days, T/C ratio of 131.37 %, and enhancement of longevity by 24 %. Interestingly, the plant extract at the highest dose, 500 mg/kg bwt, showed diminished efficacy, enhancing longevity only by 16 %.

**Table 2:** Survival values of mice transplanted with Dalton's lymphoma ascites.

Test group	MST	AST	IMLS	IALS	T/C	%T/C
Control	25.50	18.33	–	–	–	–
Doxorubicin	41.00	28.17	60.78	53.68	01.61	160.78
IKM 100	28.00	19.67	09.80	7.311	01.09	109.80
IKM 250	33.50	22.83	31.37	24.55	01.32	131.37
IKM 500	32.50	23.16	27.45	26.39	01.27	127.45

Control consisted of untreated mice bearing tumour; doxorubicin at 20 mg/kg weight; IKM100, IKM250, IKM500: treatment with *Ilex khasiana* Purkay. methanol extract at the doses of 100, 250, and 500 mg/kg bwt respectively; AST: average survival time; IMLS: increase in mean life span; IALS: increase in average life span; MST: median survival time; T/C: treatment-control ratio.

**Fig. 3:**
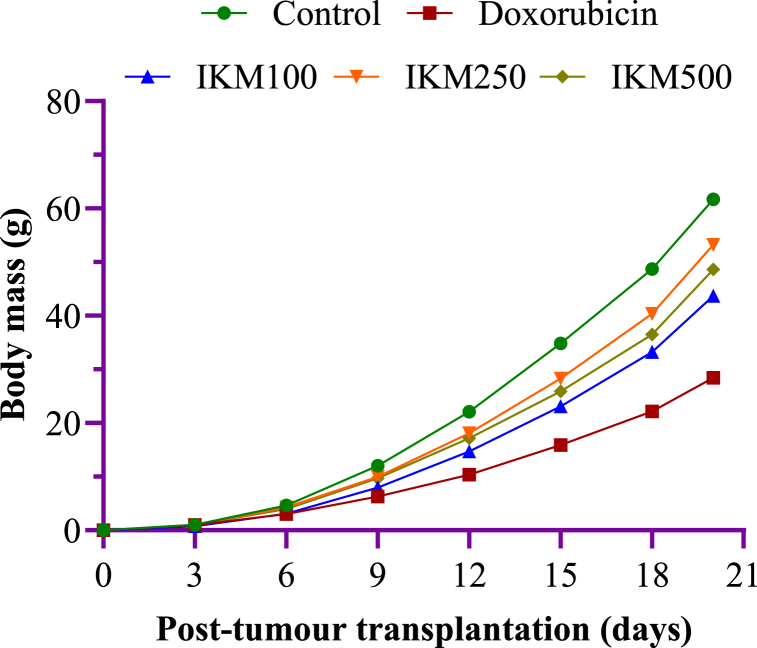
Variation of body mass in mice transplanted with Dalton's lymphoma ascites. Measurements were taken every three days for 20 days. Control: tumour-bearing untreated mice; doxorubicin at 20 mg/kg bwt; IKM100, IKM250, IKM500: treatment with *Ilex khasiana* Purkay. methanol extract at the doses of 100, 250, and 500 mg/kg bwt respectively.

An increase in the body mass following tumour induction that was due to tumour proliferation and progression was observed in all the mice. A three-day interval measurement showed that weight gain steeply increased and reached 60 % (60 g from the base weight of 0 g) in the untreated control mice as shown in Fig. 3. Gradual weight gain was observed in all the drug and plant extract treated groups. Doxorubicin showed the most potent inhibition of weight gain at 28 %. As with the survival parameters, *I. khasiana* extract showed inhibitory effect on the weight gain at all concentrations tested, but a dosage of 250 mg/kg bwt showed highest efficacy.

### 3.4 Detoxification activities

**Fig. 4:**
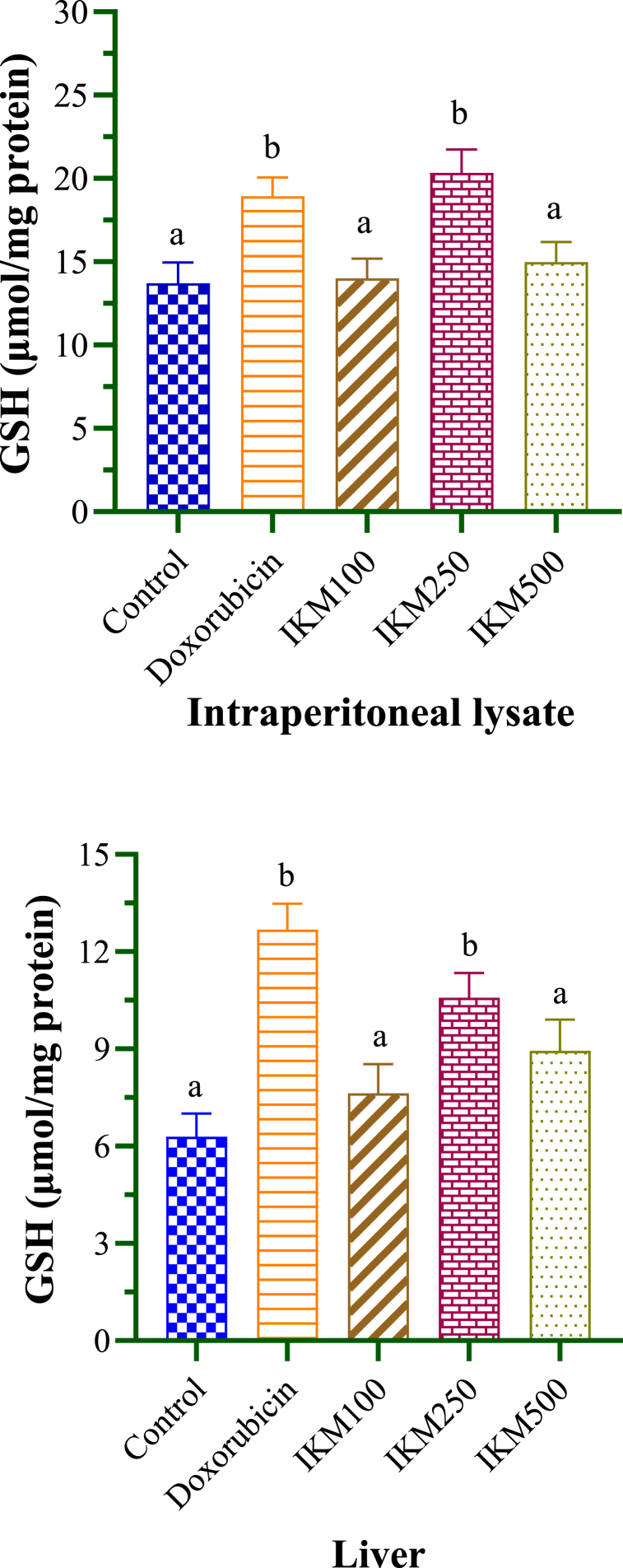
Glutathione (GSH) levels from the intraperitoneal lysate and liver of mice transplanted with Dalton's lymphoma ascites. Small letters on the bars denote comparisons between groups; same letters are significantly different at *p* < 0.05, and different letters, not significant. Control: tumour-bearing untreated mice; doxorubicin at 20 mg/kg bwt; IKM100, IKM250, IKM500: treatment with *Ilex khasiana* Purkay. methanol extract at the doses of 100, 250, and 500 mg/kg bwt respectively.

**Fig. 5:**
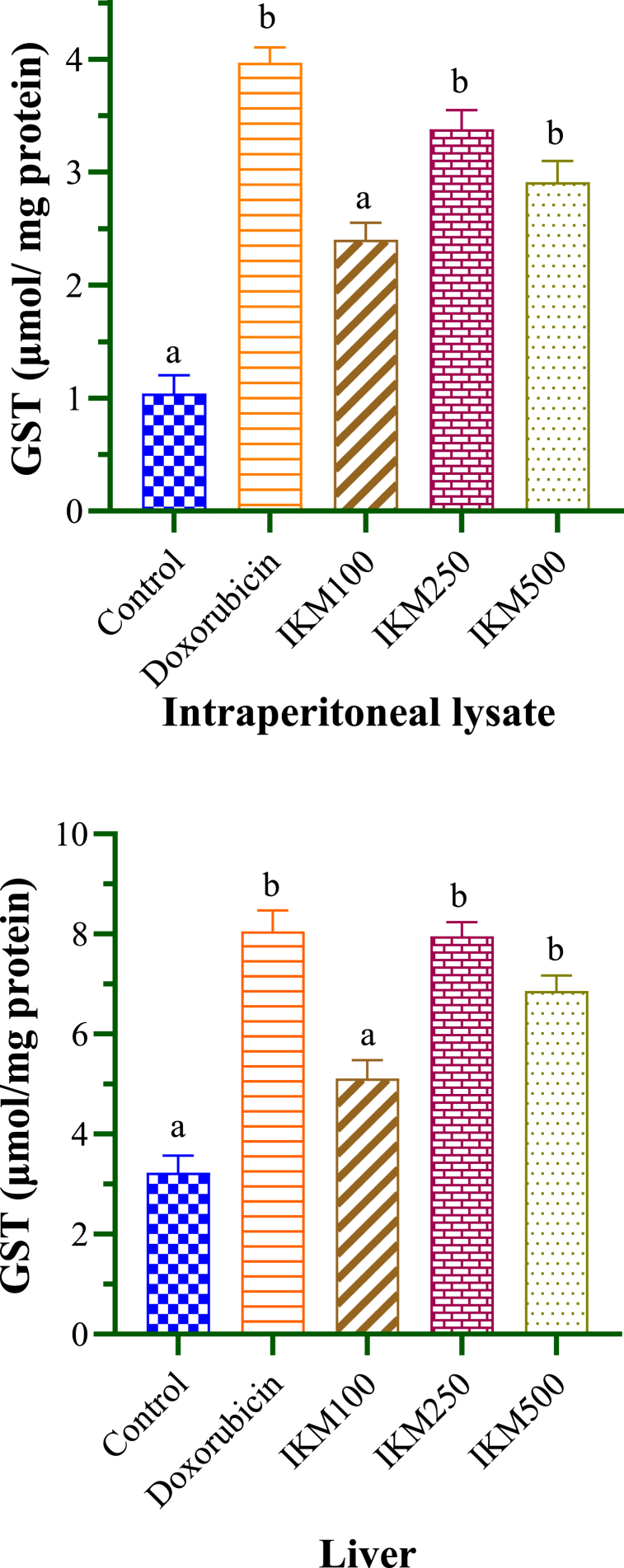
Glutathione S-transferase (GST) levels from the intraperitoneal lysate and liver of mice transplanted with Dalton's lymphoma ascites. Small letters on the bars denote comparisons between groups; same letters are significantly different at *p* < 0.05, and different letters, not significant. Control: tumour-bearing untreated mice; doxorubicin at 20 mg/kg bwt; IKM100, IKM250, IKM500: treatment with *Ilex khasiana* Purkay. methanol extract at the doses of 100, 250, and 500 mg/kg bwt respectively.

The concentrations of GSH and GST were determined from the serum samples of DLA-transplanted mice to assess the effects of doxorubicin and *I. khasiana* extract as shown in Table 3 and Fig. 4. GSH levels were estimated at 13.71 and 6.3 μmol/mg protein for the lysate and liver sample respectively in the control mice. The plant extract at 100 and 500 mg/kg bwt did not cause any significant variation. However, the extract at 250 mg/kg bwt was more effective than the drug at increasing the GSH level in the lysate, and equally effective in the liver sample. GST levels in control mice were 1.04 and 3.23 μmol/mg protein for the lysate and liver sample respectively. Only *I. khasiana* extract at 100 mg/kg bwt failed to change the enzyme level, while the 250 mg/kg bwt dosage indicated high efficacy similar to that of the drug (Fig. 5). The plant extract at 250 mg/kg bwt caused GSH increase by 19 % in the lysate and 25 % in the liver sample, and GST increase by 53 % in the lysate and 41 % in the liver sample.

**Table 3:** Glutathione (GSH) and glutathione S-transferase (GST) levels in mice transplanted with Dalton's lymphoma ascites.

Test group	GSH (μmol/mg protein)		GST (μmol/mg protein)	
	Lysate	Liver	Lysate	Liver
Control	13.71 ± 1.26^a^	6.30 ± 0.70^a^	1.04 ± 0.16^a^	3.23 ± 0.34^a^
Doxorubicin	18.95 ± 1.12^b^	12.68 ± 0.80^b^	3.97 ± 0.13^b^	8.05 ± 0.42^b^
IKM 100	14.01 ± 1.19^a^	7.63 ± 0.90^a^	2.40 ± 0.15^a^	5.11 ± 0.37^a^
IKM 250	20.34 ± 1.40^b^	10.58 ± 0.76^b^	3.38 ± 0.17^b^	7.95 ± 0.29^b^
IKM 500	14.98 ± 1.20^a^	08.95 ± 0.96^a^	2.91 ± 0.19^b^	6.86 ± 0.31^b^

Data in means ± standard error of the means. One-way analysis of variance (ANOVA) was used to determined significant difference. Small letters in superscripts denote comparisons between groups; same letters are significantly different at *p* < 0.05, and different letters, not significant. Control: untreated mice; doxorubicin at 20 mg/kg weight; IKM100, IKM250, IKM500: treatment with *Ilex khasiana* Purkay. methanol extract at the doses of 100, 250, and 500 mg/kg bwt respectively.

**Fig. 6:**
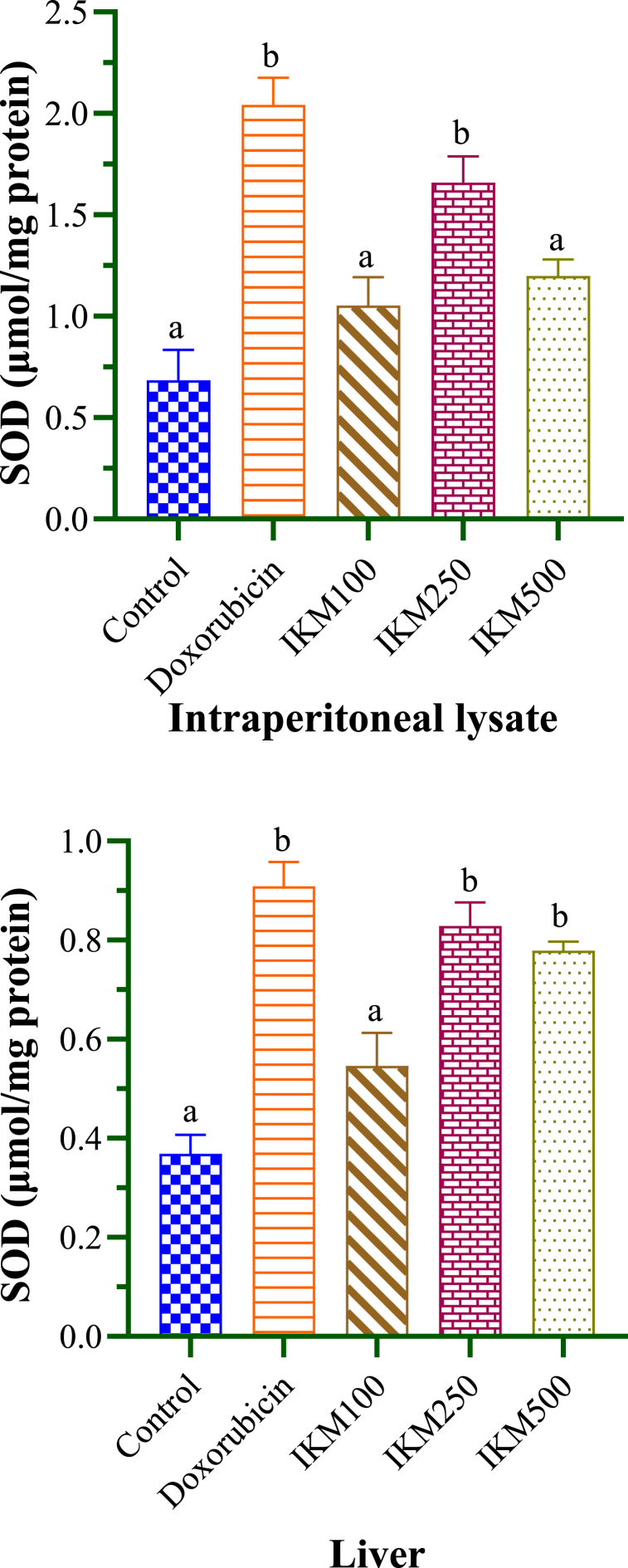
Superoxide dismutase (SOD) levels from the intraperitoneal lysate and liver of mice transplanted with Dalton's lymphoma ascites. Small letters on the bars denote comparisons between groups; same letters are significantly different at *p* < 0.05, and different letters, not significant. Control: tumour-bearing untreated mice; doxorubicin at 20 mg/kg bwt; IKM100, IKM250, IKM500: treatment with *Ilex khasiana* Purkay. methanol extract at the doses of 100, 250, and 500 mg/kg bwt respectively.

**Fig. 7:**
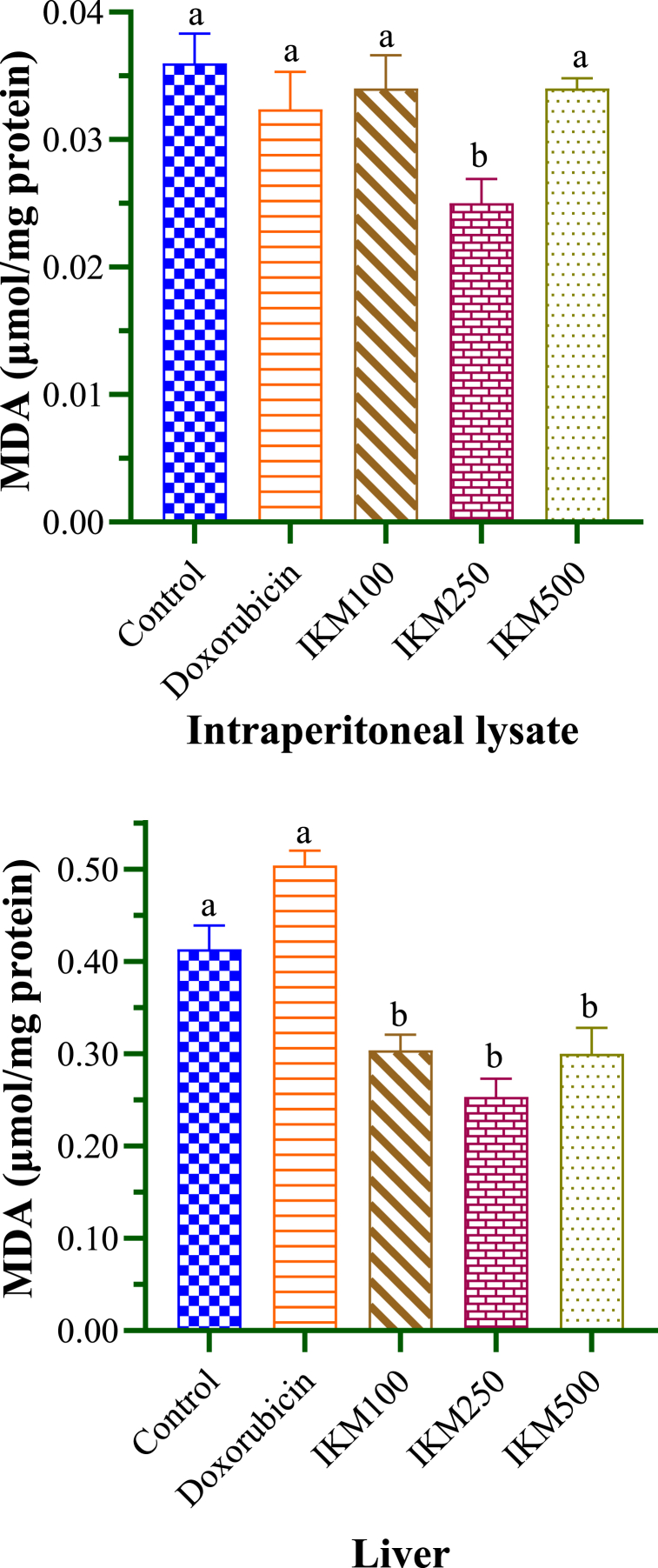
Lipid peroxidation expressed as the amount of malondialdehyde (MDA) from the intraperitoneal lysate and liver of mice transplanted with Dalton's lymphoma ascites. Small letters on the bars denote comparisons between groups; same letters are significantly different at *p* < 0.05, and different letters, not significant. Control: tumour-bearing untreated mice; doxorubicin at 20 mg/kg bwt; IKM100, IKM250, IKM500: treatment with *Ilex khasiana* Purkay. methanol extract at the doses of 100, 250, and 500 mg/kg bwt respectively.

SOD and MDA concentrations measured from experimental mice are given in Table 4. DLA-transplanted mice in control group showed SOD of 0.69 and 0.37 μmol/mg protein in the lysate and liver sample respectively. Among the different dosages of *I. khasiana* extract, only the 250 mg/kg bwt resulted in significant increase in SOD level, which was by 41 % in the lysate and 38 % in the liver sample (Fig. 6). MDA of control mice was detected at 0.036 and 0.413 μmol/mg protein in the lysate and liver sample respectively. The plant extract was effective in reducing the MDA level, especially at 250 mg/kg bwt, there was a decrease by 18 % in the lysate and by 24 % in the liver sample respectively (Fig. 7).

**Table 4:** Superoxide dismutase (SOD) and malondialdehyde (MDA) levels in mice transplanted with Dalton's lymphoma ascites.

Test group	SOD (μmol/mg protein)		MDA (μmol/mg protein)	
	Lysate	Liver	Lysate	Liver
Control	0.69 ± 0.15^a^	0.37 ± 0.04^a^	0.036 ± 0.0023^a^	0.413 ± 0.026^a^
Doxorubicin	2.04 ± 0.13^b^	0.91 ± 0.05^b^	0.032 ± 0.0029^a^	0.504 ± 0.016^a^
IKM 100	1.05 ± 0.14^a^	0.55 ± 0.07^a^	0.034 ± 0.0026^a^	0.304 ± 0.016^b^
IKM 250	1.66 ± 0.13^b^	0.83 ± 0.05^b^	0.025 ± 0.0019^b^	0.253 ± 0.020^b^
IKM 500	1.20 ± 0.08^a^	0.78 ± 0.02^b^	0.025 ± 0.0019^a^	0.300 ± 0.028^b^

Data in means ± standard error of the means. Small letters in superscripts denote ANOVA comparisons between groups; same letters are significantly different at *p* < 0.05, and different letters, not significant. Control: untreated mice; doxorubicin at 20 mg/kg weight; IKM100, IKM250, IKM500: treatment with *Ilex khasiana* Purkay. methanol extract at the doses of 100, 250, and 500 mg/kg bwt respectively.

### 3.5 Serum protein profile

**Fig. 8:**
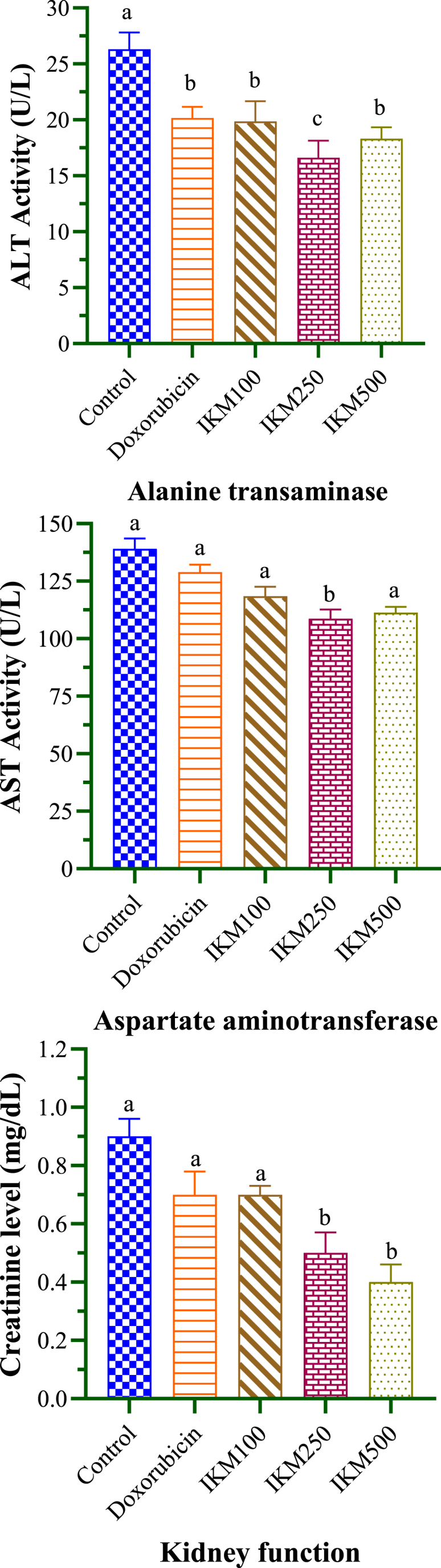
Serum profile of mice transplanted with Dalton's lymphoma ascites indicating the kevels of liver enzymes, alanine transaminase and aspartate aminotransferase, and kidney waste, creatinine. Small letters on the bars denote comparisons between groups; same letters are significantly different at *p* < 0.05, and different letters, not significant. Control: tumour-bearing untreated mice; doxorubicin at 20 mg/kg bwt; IKM100, IKM250, IKM500: treatment with *Ilex khasiana* Purkay. methanol extract at the doses of 100, 250, and 500 mg/kg bwt respectively.

Serum levels of liver enzymes and creatinine of DLA-transplanted mice are given in Table 5 and Fig. 8. Alanine transaminase and aspartate aminotransferase were determined at 26.30 and 139.12 U/L respectively in the control mice. All the dosages of *I. khasiana* extract and doxorubicin caused significant reduction of alanine transaminase. The plant extract at 250 mg/kg bwt was the most effective, causing a 23 % decrease. Of all the tested groups, only the plant extract at 250 mg/kg bwt caused significant reduction of aspartate aminotransferase, that is by 12 %. Control mice showed creatinine level of 0.9 U/L. Only the plant extract at 250 and 500 mg/kg bwt caused creatinine reduction by 28 % and 38 % respectively.

**Table 5:** Levels of serum enzymes, alanine transaminase and aspartate aminotransferase, and kidney waste, creatinine in mice transplanted with Dalton's lymphoma ascites.

Test group	Alanine transaminase (U/L)	Aspartate aminotransferase (U/L)	Creatinine (mg/dL)
Control	26.30 ± 1.52^a^	139.12 ± 1.76^a^	0.09 ± 0.06^a^
Doxorubicin	20.17 ± 0.98^b^	128.88 ± 2.32^a^	0.07 ± 0.08^a^
IKM 100	19.85 ± 1.80^b^	110.49 ± 2.99^a^	0.07 ± 0.03^a^
IKM 250	16.61 ± 1.54^c^	108.66 ± 2.31^b^	0.05 ± 0.07^b^
IKM 500	18.32 ± 1.02^b^	111.30 ± 2.54^a^	0.04 ± 0.06^b^

Data in means ± standard error of the means. Small letters in superscripts denote ANOVA comparisons between groups; same letters are significantly different at *p* < 0.05, and different letters, not significant. Control: untreated mice; doxorubicin at 20 mg/kg weight; IKM100, IKM250, IKM500: treatment with *Ilex khasiana* Purkay. methanol extract at the doses of 100, 250, and 500 mg/kg bwt respectively.

### 3.6 DNA damage

**Fig. 9:**
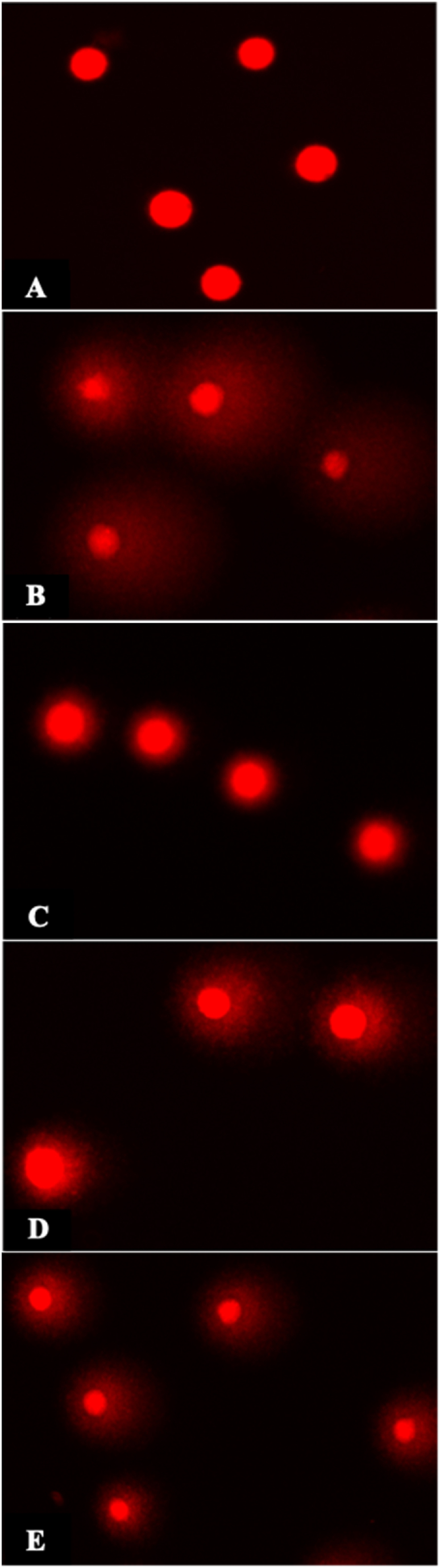
Fluorescent micrographs of Dalton's lymphoma ascites transplanted in mice under different experimental conditions from comet assay: (A) negative control; (B) positive control, i.e., treated with doxorubicin at 20 mg/kg bwt; (C), (D) and (E), treated with *Ilex khasiana* Purkay. methanol extract at the doses of 100, 250, and 500 mg/kg bwt respectively. Tail lengths and Olive moment were determined from the area of the comets surrounding the cells that indicate DNA fragmentations.

**Fig. 10:**
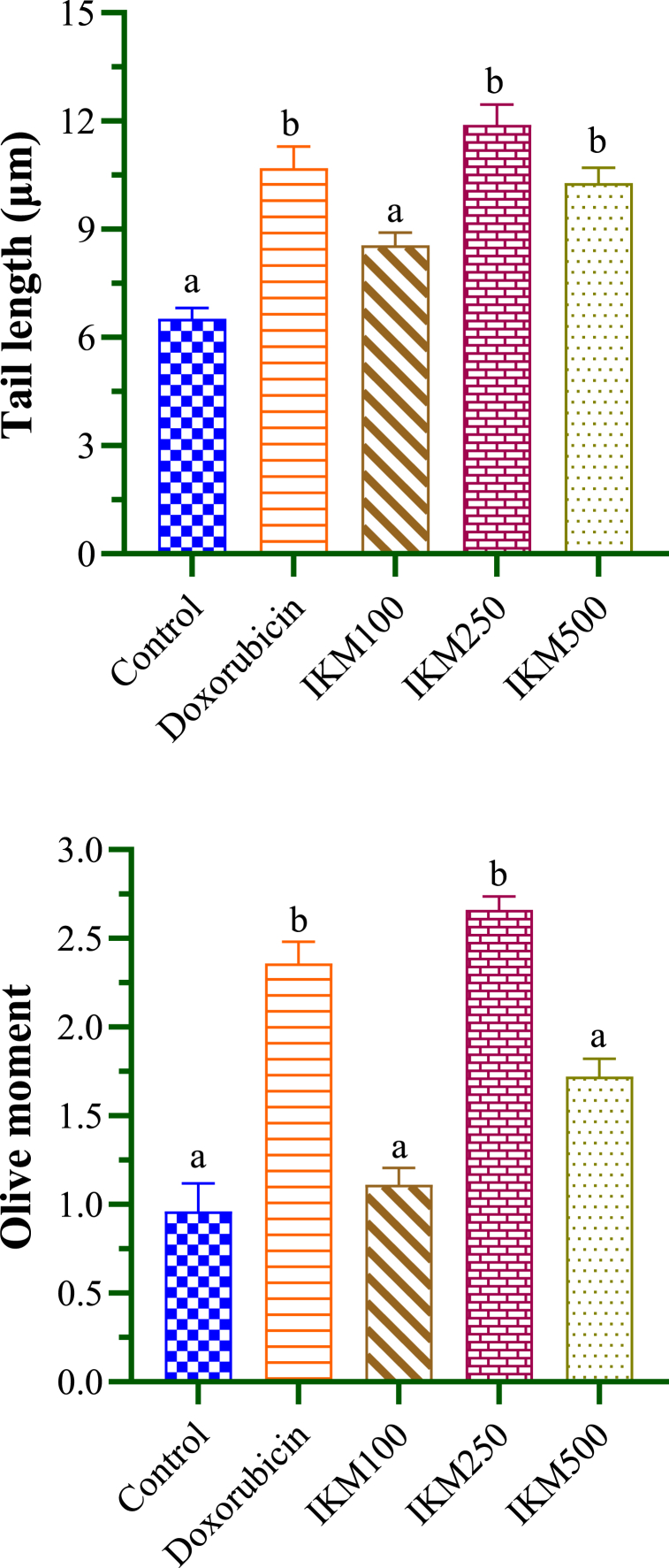
Comparison of DNA damage from comet assay of Dalton's lymphoma ascites transplanted in mice. Small letters on the bars denote comparisons between groups; same letters are significantly different at *p* < 0.05, and different letters, not significant. Control: tumour-bearing untreated mice; doxorubicin at 20 mg/kg bwt; IKM100, IKM250, IKM500: treatment with *Ilex khasiana* Purkay. methanol extract at the doses of 100, 250, and 500 mg/kg bwt respectively.

The compactness of DNA organization and changes after comet assay are shown Fig. 9. Cells in control showed fine margins (Fig. A). Uniform comets were seen on cells treated with doxorubicin (Fig. 9B). Among the *I. khasiana* extracts, the largest comets were produced by 250 mg/mL (Fig. 9C), followed by 500 mg/mL (Figs. 9D) and 100 mg/mL (Fig. 9E). DNA damage in cancer cells is reflected by the degree of the trails, tail length and Olive moment which are detectable due to DNA double strand breaks. The tumour lysate from the DLA-transplanted mice showed a tail length of 2.25 μm and an Olive moment of 0.96 (Table 6). Comparisons of the tail length and Olive moment are given in Fig. 10. The plant extract at 100 mg/kg bwt did not affect the DNA integrity, and at 500 mg/kg bwt, it did not significantly alter the Olive moment although it enhanced the tail length by 23 %. The plant extract at 250 mg/kg bwt was the most effective, even more so than that of doxorubicin, causing 28 % and 47 % increase in the tail length and Olive moment respectively.

**Table 6:** Measurements of DNA damage in Dalton's lymphoma ascites in mice from comet assay.

Test group	Tail length (μm)	Olive moment
Control	02.52 ± 0.30^a^	0.96 ± 0.160^a^
Doxorubicin	10.70 ± 0.60^b^	2.36 ± 0.120^b^
IKM 100	08.56 ± 0.34^a^	1.11 ± 0.097^a^
IKM 250	11.89 ± 0.57^b^	2.66 ± 0.076^b^
IKM 500	10.28 ± 0.43^b^	1.72 ± 0.100^a^

Data in means ± standard error of the means. Small letters in superscripts denote ANOVA comparisons between groups; same letters are significantly different at *p* < 0.05, and different letters, not significant. Control: untreated mice; doxorubicin at 20 mg/kg weight; IKM100, IKM250, IKM500: treatment with *Ilex khasiana* Purkay. methanol extract at the doses of 100, 250, and 500 mg/kg bwt respectively.

### 3.7 Computational molecular interaction

**Fig. 11:**
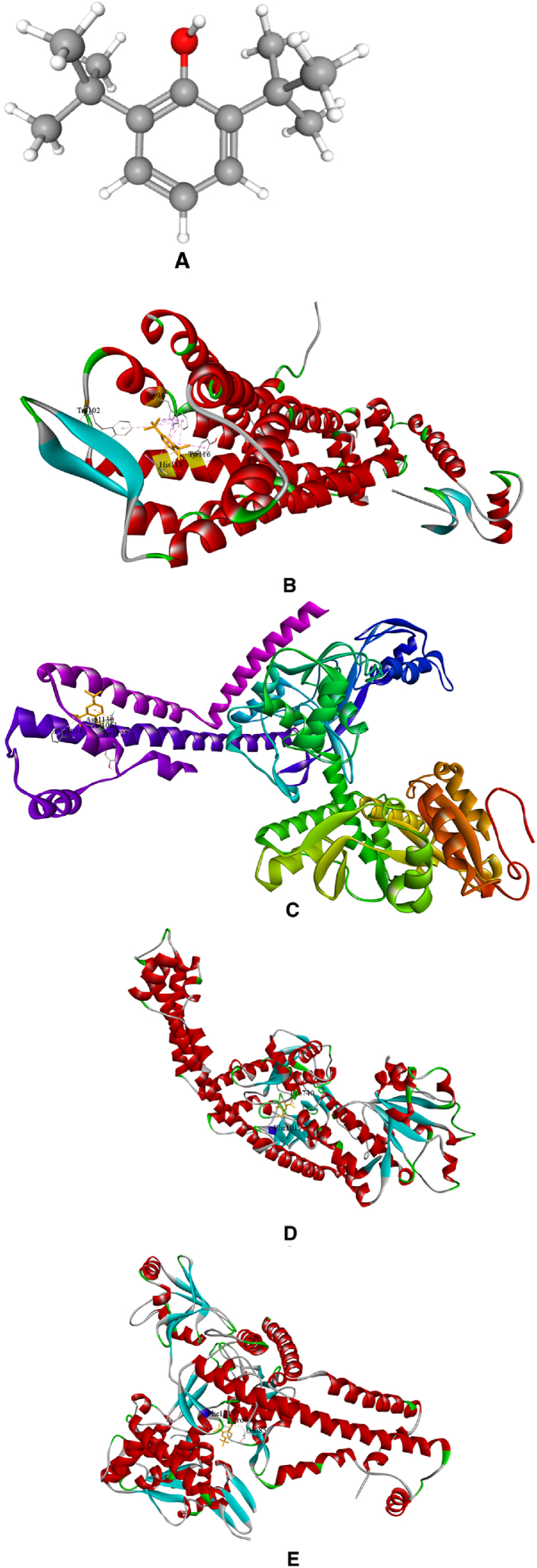

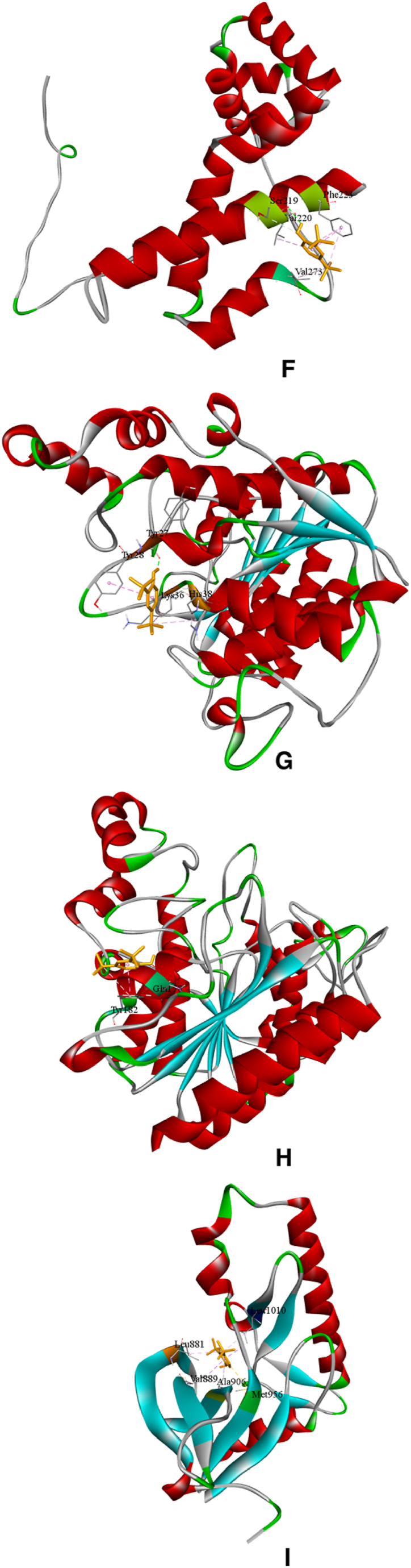

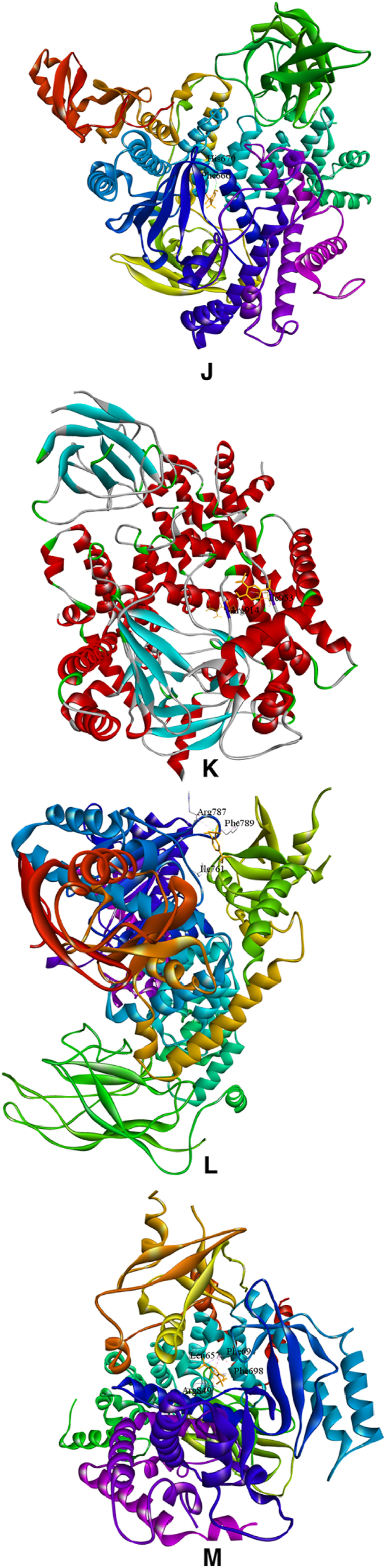

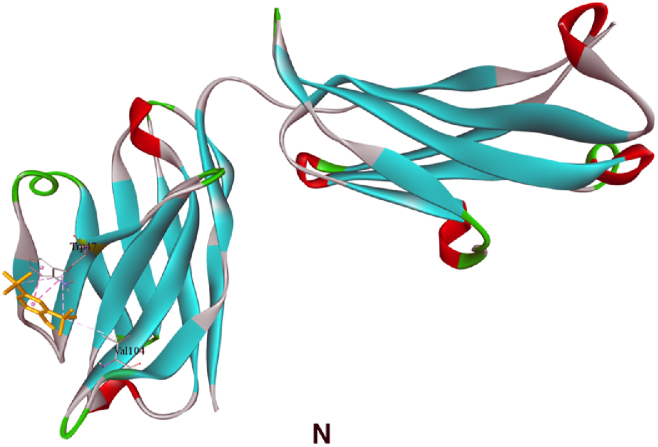
3D structures of ligand binding between 2,6-di-tert-butylphenol and different receptor proteins that are implicated in lymphoma development and anti-lymphoma drugs. (A) 2,6-Di-tert-butylphenol; (B) Chemokine receptor CXCR4; (C) DNA topoisomerase 2-alpha; (D) DNA topoisomerase 2-beta; (E) DNA topoisomerase 2-beta (different configuration); (F) histone deacetylase (HDAC) 1; (G) HDAC 2; (H) HDAC 3; (I) Janus kinase 1 (JAK1); (J) phosphoinositide 3-kinase (PI3Kɑ); (K) PI3Kβ; (L) PI3Kβ (different configuration); (M) PI3Kγ; (N) programmed cell death protein 1 (PD-1).

The principal compound, 2,6-di-tert-butylphenol, was used for simulating the probable anticancer action of *I. khasiana* extract at the molecular levels. Docking was done on the crystallographic structures of 11 receptors known for their association with lymphoma; two receptors, DNA topoisomerase II-beta and PI3Kβ, were docked in duplicates as there are fine alternative crystallographic structures for them in the protein data bank (Fig. 11A–N). The preparative molecular interaction with the grid measurements and PDB sources are depicted in Table 7. The relative binding scores and specific binding sites on each receptor are presented in Table 8. As predictive from the *in vivo* tests and DNA damage assessment, 2,6-di-tert-butylphenol showed high binding energy scores on all the receptors docked. The best binding affinity of the compound was on phosphoinositide 3-kinase (PI3Kγ) indicated by an energy scoring function of −7.5 kcal/mol, and close to its was on CXCR4 with −7.1 kcal/mol. Not unsurprisingly, even the receptor with the least binding function, PD-1 with a binding energy of −5.5 kcal/mol is still a substantial level of interaction.

**Table 7:** Molecular data set up for docking in AutoDock Vina, showing the grid size, centre position and protein data bank (www.rcsb.org) identification codes.

Receptor	Size x	Size y	Size z	Centre x	Centre y	Centre z	PDB code	Reference
Chemokine receptor (CXCR4)	68	56	104	5.528	21.754	41.207	3OE6	[32]
DNA topoisomerase II-alpha	78	108	74	34.839	51.801	15.908	4FM9	[33]
DNA topoisomerase II-beta	90	100	68	28.344	114.033	68.149	3QX3	[34]
DNA topoisomerase II-beta	106	64	94	32.551	−49.287	−28.5	5ZAD	[35]
Histone deacetylase 1 (HDAC1)	66	70	40	−62.981	18.93	4.152	4BKX	[36]
HDAC2	58	56	60	14.057	−20.339	−1.928	4LY1	[37]
HDAC3	56	56	62	42.746	54.793	22.481	4A69	[38]
Janus kinase 1 (JAK1)	54	54	40	11.056	9.627	−10.276	3EYG	[39]
Phosphoinositide 3-kinase (PI3Kɑ)	104	82	96	−4.494	−22.099	27.415	4JPS	[40]
PI3Kβ	100	82	86	−19.925	65.412	82.332	4BFR	[41]
PI3Kβ	78	98	96	31.788	−47.653	8.955	2Y3A	[42]
PI3Kγ	86	76	72	29.872	0.372	25.787	4ANW	[43]
Programmed cell death protein 1 (PD-1)	66	44	64	−21.608	3.694	−47.197	6JJP	[44]

**Table 8:** Molecular docking scores of 2,6-di-tert-butylphenol binding on different receptors associated with lymphoma and the specific binding sites.

Receptor	PDB code	Energy score (kcal/mol)	Amino acid residue	Amino acid position
Chemokine receptor (CXCR4)	3OE6	−7.1	Tryptophan	94
			Tryptophan	102
			Histidine	113
			Tyrosine	116
DNA topoisomerase II-alpha	4FM9	−6.2	Glutamine	1051
			Phenylalanine	1054
			Tyrosine	1127
			Aspartic acid	1130
DNA topoisomerase II-beta	3QX3	−6.2	Proline	740
			Phenylalanine	1019
DNA topoisomerase II-beta	5ZAD	−6.2	Proline	740
			Glutamate	855
			Phenylalanine	1019
Histone deacetylase 1 (HDAC1)	4BKX	−5.3	Serine	219
			Valine	220
			Phenylalanine	223
			Valine	273
HDAC2	4LY1	−5.6	Tyrosine	28
			Tyrosine	27
			Lysine	36
			Histidine	38
HDAC3	4A69	−5.5	Glutamine	178
			Tyrosine	182
Janus kinase 1 (JAK1)	3EYG	−6.0	Leucine	881
			Valine	889
			Alanine	906
			Methionine	956
			Leucine	1010
Phosphoinositide 3-kinase (PI3Kɑ)	4JPS	−6.8	Phenylalanine	666
			Histidine	670
PI3Kβ	4BFR	−5.9	Arginine	914
			Isoleucine	953
PI3Kβ	2Y3A	−6.2	Isoleucine	761
			Arginine	787
			Phenylalanine	789
PI3Kγ	4ANW	−7.5	Leucine	657
			Phenylalanine	694
			Phenylalanine	698
			Arginine	849
Programmed cell death protein 1 (PD-1)	6JJP	−5.5	Tryptophan	47
			Valine	104

## 4 Discussion

Consumption of *I. khasiana* leaves appeared to be phenomenally safe, as the methanol extract was comfortably tolerated by experimental mice. Even the highest toxicity dose, i.e., 2000 mg/kg bwt, recommended by OECD did not instigate a marginal abnormal behaviour, implying that the exact LD_50_ would be over the maximum dosage tested. By default, an LD_50_ at a dosage of 20 g/kg bwt is a typical threshold for toxicity [49], thus, the plant extract should be considered highly non-toxic and safe. 2,6-Di-tert-butylphenol at a relative abundance of 100 % and probability 0f 73 % was by far the major chemical constituent, suggesting that the biological activities, if not all, of the plant extract are due to it. The alkylated phenol has been known from various plants and is an established antioxidant molecule, the property for which it has attracted several chemical and pharmacological investigations [50,51]. Most importantly, it is used as a base compound by incorporating it to synthetic compounds as a functional moiety in the preparation of several bioactive molecules that are validated to have anticancer activity against different cell lines [[52], [53], [54]]. Two other major compounds detected in *I. khasiana* are known to have important biological activities. Desulphosinigrin is attributed as one of the phytocompounds having anticancer, antimicrobial [55], and antiprotozoal activities [56]. Palmitic acid is demonstrated for its antimicrobial, antioxidant [57,58], and anticancer activities [59].

Evaluation of longevity of experimental animals is a well-grounded criterion for anticancer activity and potency of test materials [60]. The prolongation of survival time in cancer subjects is a fundamental goal of anticancer agents [61]. DLA is a highly malignant and aggressive tumour T lymphocyte, and thus, is a suitable model for determining anticancer susceptibility and drug responses [62]. Our data indicated a high antitumour progression in mice following treatment with *I. khasiana* extract sharply declined; especially at 250 mg/kg bwt, longevity increased by 33 %. All the test materials, except the plant extract at 100, showed a T/C ratio higher than 120 %, the base limit demarcated by the US National Cancer Institute guideline for mice leukemia [63]. The plant extract, therefore, is evidently implied as having appreciable anticancer potency against lymphoma. An increase in the body mass following tumour induction was observed in all the mice, the general fact that is known due to tumour proliferation and progression [64]. Doxorubicin is an efficacious drug in causing tumour regression, and thereby causing weight reduction in malignancy due to DLA [65]. Parallel dosage-dependent effect of tumour inhibition was noted in mice treated with the plant extract.

The anticancer property of *I. khasiana* is fortified by haematological profiles of the treated mice. GST is member of a family of detoxifying enzymes that catalyses the conjugation of glutathione GSH with xenobiotics; thus, making the two proteins reliable indicators of anticancer effects [66]. GSH and GST are critical in cancer and autoimmune cells as they are indispensable proteins for maintaining cellular redox homeostasis, such that a signature anticancer effect is increasing the cellular productions of the proteins so as to check aberrant redox state due to tumour development or immune disorders [67]. This elevated condition of GSH and GST is a signature of effective anticancer activity that can be conveniently inferred when a test material induces enhanced levels of the proteins. SOD is a mitochondrial matrix enzyme having strong antioxidant potency in eliminating superoxide radical (O2^•–^) [68]. Anticancer effect is reliably reflected by the ability to raise the circulating levels of SOD as seen in this study. Lipid peroxidation is a free radical-mediated cell conversion of lipids to peroxide and hydroperoxide derivative. MDA is a final product of such lipid peroxidation and is highly reactive with high binding affinity for DNA molecules. Therefore, MDA level is a robust physiological marker of oxidative stress, cancer conditions and anticancer effects [69]. Suppression of MDA in the present data is what is to be expected of anticancer molecules. The two liver enzymes, alanine transaminase and aspartate aminotransferase, are used as clinical predictor of liver damage and cancer as they accumulate in diseased conditions [70], making the level of clearance from the circulatory system a diagnostic parameter of anticancer activity [71] Creatinine is a clinical marker of metabolic perturbations useful for detecting metabolic disorders and cancer [72], and hence, anticancer activity [73].

A peculiar observation was that the plant extract was most potent at the medium dosage, i.e., 250 mg/mL bwt, in some of the key parameters of anticancer activity, while the effectiveness consistently waned at the highest dose, i.e., 500 mg/kg bwt. However, it is not an improbable pharmacological condition as many drug molecules have their threshold efficacy at higher doses so that at certain point, drug responsiveness and molecular effects decreased to little or no activity [74]. Such drug insensitivity, the phenomenon known as hormesis, has been lately a critical concern in clinical managements of microbial infections and cancers [75].

Comet assay is based on a direct observation that DNA damages can be visualised as formation of fragments from a fixed point and that the fragments under electrophoresis form trails that can be conveniently measured using fluorescent microscopy [34]. In a nutshell, the greater the area of the comets, the higher the values of the breaks, and thereby, higher is the potency of the anticancer agent [76]. It is a remarkable observation that *I. khasiana* extract at 250 mg/kg bwt was exceptionally powerful in causing DNA damage, surpassing even the anticancer drug used. The plant extract produced 5.3 % and 5.9 % higher DNA fragmentation than doxorubicin measured from the comet tail length and Olive moment respectively.

The target proteins selected for molecular docking are the most universal receptors ascribed to the development of and drug action on T-cell lymphoma [77,78]. The main bioactive compound of *I. khasiana* extract, 2,6-di-tert-butylphenol was found to have high binding affinity to all the receptors. Although it should be noted that computational scores do not necessarily represent the actual molecular interaction, or the exact binding fitness, it is nonetheless appreciated the more the values deviate towards negative functions, the more likely the chances of precise binding [79]. Even at the least binding energy, i.e., −5.5 kcal/mol against PD-1, the compound displayed an excellent binding affinity. Highest binding affinities were seen on CXCR4 and PI3Kγ. CXCR4 is a member of the G protein-coupled receptors superfamily having substantiated roles in angiogenesis, metastasis, and drug resistance. It is one of the key focuses in cancer drug development [80]. PI3Kγ is within the network one of the best characterised enzymatic pathways involved in lymphoma emergence and progression, and thus, its inhibition is one of the major targets in anticancer effects on lymphomas [81,82]. From the overall observation, it becomes evident that *I. khasiana*, by and large, possesses anticancer property as is claimed in the Mizo traditional medicine. As a critically endangered species, the findings are compelling advocacy for measures to initiate conservation to make any further meaningful research on this plant.

## 5 Conclusion

The methanol extract of *I. khasiana* leaves contains 2,6-di-tert-butylphenol as the principal chemical component. A series of *in vivo* tests revealed that the plant extract has low toxicity but high cytotoxicity against Dalton's lymphoma ascites in mice. The plant extract, especially at 250 mg/kg bwt, was comparable to that of the reference drug doxorubicin in the different parameters used for anticancer assay. It significantly increased the lifespan of DLA-transplanted mice, while reducing the body mass due to tumour development. It increased the levels of glutathione and glutathione S-transferase, as well as superoxide dismutase activity, while reducing lipid peroxidation. It was more efficacious than doxorubicin at promoting the clearance and elimination of liver enzymes, alanine transaminase and aspartate aminotransferase, as well as a kidney waste, creatinine. The plant's ability to induce DNA damage was evidence by the increased comet formation under gel electrophoresis. Pharmacological modelling of 2,6-di-tert-butylphenol for its potential anticancer mechanism against the major target proteins in lymphoma showed effecting binding and interaction with high affinity to the key proteins. Further insight into the molecular interactions, precise mechanism of action, and pharmaceutical tests are warranted by the present findings. Furthermore, as a critically endangered species, *I. khasiana* deserves further biological attention and conservation to fully explore its potential usefulness in pharmaceutical development.

## CRediT authorship contribution statement

**Charles Lalnunfela:** Writing – original draft, Investigation, Formal analysis, Data curation. **Pawi Bawitlung Lalthanpuii:** Visualization, Validation, Methodology, Formal analysis. **Hmar Tlawmte Lalremsanga:** Supervision, Resources, Project administration. **Zothansiama:** Supervision, Methodology, Conceptualization. **Chhaihlo Lalmuansangi:** Validation, Investigation, Formal analysis. **Mary Zosangzuali:** Investigation, Formal analysis. **Nachimuthu Senthil Kumar:** Resources, Project administration, Funding acquisition. **Tochhawng Lalhriatpuii:** Supervision, Resources, Project administration, Conceptualization. **Kholhring Lalchhandama:** Writing – review & editing, Supervision, Resources, Methodology, Funding acquisition, Formal analysis, Conceptualization.

## Ethics approval

This study was reviewed and approved by the Institutional Animal Ethics Committee of the Regional Institute of Paramedical and Nursing Sciences, Mizoram, India, with the approval number: IAEC/RIPANS/27, dated July 5, 2018.

## Data availability

The data used to support the findings of this study are included in the article.

## Declaration of competing interest

The authors declare the following financial interests/personal relationships which may be considered as potential competing interests:K. Lalchhandama reports equipment, drugs, or supplies and travel were provided by India Ministry of Science & Technology Department of Biotechnology. If there are other authors, they declare that they have no known competing financial interests or personal relationships that could have appeared to influence the work reported in this paper.
